# Prevalence of COVID-19 Pandemic, Self-Esteem and Its Effect on Depression Among University Students in Saudi Arabia

**DOI:** 10.3389/fpubh.2022.836688

**Published:** 2022-02-08

**Authors:** Fatima M. Azmi, Habib Nawaz Khan, Aqil M. Azmi, Arooj Yaswi, Mihajlo Jakovljevic

**Affiliations:** ^1^Department of Mathematics and Sciences, College of Humanities and Sciences, Prince Sultan University, Riyadh, Saudi Arabia; ^2^Department of Statistics, University of Science and Technology, Bannu, Pakistan; ^3^Department of Computer Science, College of Computer and Information Sciences, King Saud University, Riyadh, Saudi Arabia; ^4^Department of General Studies-Psychology, College of Humanities and Sciences, Prince Sultan University, Riyadh, Saudi Arabia; ^5^Department Global Health Economics and Policy, University of Kragujevac, Kragujevac, Serbia; ^6^Department of Comparative Economics Studies, Hosei University Faculty of Economics, Tokyo, Japan

**Keywords:** COVID-19, Saudi Arabia, Rosenberg Self-Esteem Scale, Zung Self-Rating Depression Scale, university students, depression, economic crisis

## Abstract

**Background and Aims:**

University students are commonly identified as susceptible, suffering from higher anxiety, stress, and depression than the overall population. During the Corona Virus Disease pandemic (COVID), education was shifted to the virtual learning environment. Students' ambiguity regarding academic accomplishment, imminent careers, changes in social life, and other concerns all these factors played a role in amplifying their stress levels, anxiety, and depression worldwide. This study investigates university students' self-esteem and depressions after they have been online learning for over 1 year due to the COVID-19 pandemic in Saudi Arabia.

**Methods:**

For this research, an adapted questionnaire of Rosenberg (Self-Esteem Scale) and Zung (Self-Rating Depression Scale) was used to get the responses of the participants of public and private universities in Saudi Arabia during March-April 2021. We received a total of 151 valid responses from respondents. For data analysis, we used descriptive statistics, ANOVA, multiple regression and binary logistic regression.

**Findings:**

The results showed that 75% of the students experienced different levels of depressions, with half (37.5%) having moderate to extreme levels of depression. A total of 41% of students experienced low self-esteem (38% females and 45% males). The regression results indicated depressive symptoms for low self-esteem. Furthermore, results of logistic regression showed that high self-esteem reduces the chances of getting depressive symptoms by 17%. The depressive symptoms were higher in female students than their male counterparts; furthermore, males experienced depressive symptoms less than females by 38%.

**Conclusions:**

Based on the current research results, it is concluded that the presence of the COVID-19 pandemic has dramatically increased the depressive symptoms in students, especially in female students. The findings suggested instant consideration and support for students. It is also suggested to the quest for potential managing policies that have been known and effective during the pandemic. Moreover, training should be provided for students to shift their educational experience mindset to an adaptive mindset, which can help them adapt to the new ways of learning and education.

## Introduction

The COVID-19 pandemic outbreak is considered the foulest pandemic in this era. On March 11, 2020, the World Health Organization (WHO) declared Coronavirus a pandemic. Due to the increasing number of COVID-19 cases, different countries exercised partial and complete lockdowns, with strict shutdowns estimated to have saved ~3 million lives across 11 European countries (1). Saudi Arabia has no exception in this regard, and the ministry of health (MOH) applied numerous measures to control and combat the spread of the virus. Besides quarantine of infected people, using masks, keeping social distance, most schools and universities were closed, and online teaching methods were adopted for education. The pandemic had profound effects on mammoth-sized populations and health systems such as the one of India (2). At the same time, disruption of trade routes and imposed barriers to the movement of people and goods have triggered a global economic crisis (3).

Worldwide, the COVID-19 pandemic kept about 300 million school students at home (4). Despite contradictory opinions about the effectiveness of keeping children home (5), school closure decisions were considered the safest way globally. As a result, students' educational journeys have been unpredictably and severely troubled to contain the virus.

Many studies covered the long and short-run effects of the pandemic on the social as well as psychological wellbeing of the population (6). A significant number of papers support that the COVID-19 pandemic has intensely obstructed people's behavior and mental health (7, 8). Some studies suggest otherwise (9). Mental health hotlines in the USA had experienced 1,000% increases during the time when more individuals were under lockdown due to the pandemic (10).

Students are severely affected by COVID-19 because of ambiguity concerning academic achievement, social life during college, and future careers, among other concerns (11). In addition to the risk of infection and possible death, people around the globe received substantial psychological pressure from the pandemic (12–14). Even before the pandemic, students worldwide have experienced augmented anxiety levels, depressive moods, psychosomatic problems, and lack of self-esteem (15). Thus, students may require extra resources and services to manage physical and mental health impacts due to increased anxiety, stress, and depression.

A recent analysis highlighted some of the known psychological effects of COVID-19 on students (16). For example, many students feel augmented stress levels, depressive symptoms, and anxiety as a result of transformed teaching delivery and indecision of university education, added to it technological worries of virtual teaching, being away from home, decreased family income, social isolation, and future employment, all these influences were observed in universities across the globe (11).

Due to pandemics, psychological distress, anxiety, depression, and lack of self-esteem vary from one country to another. For instance, research conducted in Italy found that 15.4% of Italian have an extremely high level of depression, 11.5% are highly anxious, and 12.6% are extremely stressed (17).

In Saudi Arabia (KSA), the psychological effect of the COVID-19 pandemic has been stated; Al-Hanawi et al. (18) indicated that 40% of the population experienced different ranges of distress due to COVID-19. Moreover, Alkhamees et al. reported that 23.6% of the Saudi general public experienced moderate to severe psychological impact (19). Higher rates were found to prevail among females, young people, and health practitioners (18, 19). A 20% level of anxiety was also found among college-level Saudi students by Amr et al. (20). The study of Al-Gelban results also confirmed depression and anxiety among high school students have depression and anxiety; 29.3% had moderate to severe anxiety levels, while 40% were moderate to severely depressed (21). At the same time, Khoshaim et al. showed that 13% of students experienced severe to extremely severe anxiety levels due to the pandemic (22). The Saudi authorities realized the escalation of psychological disorders; thus, health guidelines and messages were circulated to the public. For instance, the Saudi Center for Disease Control and Prevention CDC (23) provided a precautionary guide for social and mental health concentrated on prevention and fear and stress management during the pandemic.

Since March 8, 2020, education in Saudi Arabia was shifted to online teaching due to the COVID-19 pandemic. It has been more than 1 year since schools and universities have been closed and education became virtual. This new teaching and learning environment became an extraordinary and unexpected experience for both teachers and students. We can expect that the new learning and assessment situation combined with other factors of living in the protective environment due to the COVID-19 pandemic, social isolation, wearing masks, and the fear of being infected or losing a loved one to the diseases; might be related to psychological challenges for the students; all this could put tremendous pressure on them, affecting their self-esteem and causing anxiety and depression.

Depression in university students is a vital disorder that disturbs the quality of their life by causing emotional, physical, and behavioral changes and can continue into adulthood (24). The Zung Self-Rating Depression scale (ZSDS) is a 20-item self-report assessment device to measure depression levels, which Zung developed in 1965 (25). The total depression index score ranges from 25 to 100 and it has four levels of depression; normal, mild, moderate, and severe.

Self-Esteem is one of the depression-related factors that is a crucial factor for psychological interventions (26). Health research has broadly considered the association between self-esteem and depression over the past era (27, 28).

Self-esteem is defined as “a certain attitude and a perception of oneself” (29), affecting interactions and feelings toward oneself and others. Rosenberg Self-Esteem Scale (RSES) is a 10-item standardized resource which is an important tool used in clinical and research practice (30, 31). The total sum of the scores ranges from 10 to 40. Most studies that apply RSES use the mean of the total score as the primary indicator (32, 33). The RSES offers the results in two levels, low and high (26). Most studies focus on internal consistency without paying much attention to the Self-Esteem levels; only a few studies used the Self-Esteem level to compare groups of subjects (34). Studies showed that self-esteem plays a vital role in depression (35). In addition, much research studied the interplay between depression and self-esteem to find whether a causal link exists between depression and self-esteem or not (36). Recent studies showed that self-esteem predicts depression negatively (27).

In Saudi Arabia, rare studies investigated the relationship between depression and self-esteem among university students (37). To our knowledge, there is no research available to find the relationship between depression and self-esteem among the university students of Saudi Arabia during the COVD-19 pandemic. So, it is the need of educational institutes to evaluate students' psychological wellbeing.

This study investigates self-esteem and depression among university students in Riyadh during the COVID-19 pandemic. Furthermore, it explores the levels of Self-Esteem and depressions and whether low levels of self-esteem indicate depressive symptoms among university students. This research differs from the previously available research in manifolds: (i) this research tries to identify key factors affecting depression. (ii) Data generating process (DGP) is a key to determining the appropriate statistical technique to be used for statistical analysis. The tools used for analysis are used on DGP. (iii) Different methods are used for robustness results. Therefore, by understanding the current situation of self-esteem and depression among university students in Saudi Arabia, appropriate measures can be taken to help students cope with these extraordinary situations, which will help them maintain a healthy mindset rather than avoid stress (38).

## Research Methodology

### Sampling Procedure

An online survey was conducted to gather data for the current study about Self-Esteem and Depression among University students in Riyadh, which is the capital of Saudi Arabia, having over 7 million populations and an area of 1,973 square km (39). Among the numerous public and private universities, two universities were chosen for this study based on their reputation and a more significant number of enrolled students. These were King Saud University (KSU), one of the oldest public universities in Saudi Arabia, having over 55,000 enrolled students, and Prince Sultan University (PSU), a leading private institution, having over 5,500 enrolled students. In addition, the participants for the study belonged to both genders and were from different specializations and different levels of study at the university.

After receiving approval from PSU's Institutional Review Board (IRB-Ref. No. PSU IRB-2021-03-0077), a questionnaire was prepared using Survey Monkey. The questionnaire included demographic survey questions, such as gender, age, and field of study. In addition, a 10-item Rosenberg Self-Esteem Scale (RSES) was utilized (30, 31). RSES is a standardized scale, and it has been proven to be valid and reliable across many diverse samples (40); Zung's Self-Rating Depression Scale (SDS), having 20 items on four points Likert scale, was utilized to measure the depression (25, 41). Students were briefed about the nature of the survey and how to fill the survey after reading it carefully. The survey takes about 8–10 min to complete, and it was conducted with university students from March to April 2021. A total of 160 responses were received, but 151 were complete responses; 74 (49%) males and 77 (51%) females.

### Measuring Instruments

#### Rosenberg Self-Esteem Scale

In this study, the 10-item Rosenberg Self-Esteem Scale (RSES) (30, 31) is used, which is a standardized resource proven to be valid and reliable across many different samples (40). This test was translated into Arabic and was utilized among Arabic-speaking populations (41, 42).

The questionnaire consists of ten items scoured on a four-point Likert scale ranging from 1 (strongly disagree) to 4 (strongly agree). It consists of five positively worded and five negatively worded items; the negative items were reversely scored. Each item was coded from 1 to 4 and summed so that the possible sum of scores ranged from 10 to 40. Participants were considered to have a low level of self-esteem if their score was in the range 10–28 and a high level of self-esteem if their score ranged 29–40 (26). Although the teaching language in the university is English, the authors wanted to make sure that students understood the questions correctly; therefore, the RSES questionnaire was prepared in both Arabic and English side by side, the Arabic translation was used as appeared in (41, 42). In this study, Cronbach's α coefficient was 0.865, indicating high internal consistency.

#### Zung Self-Rating Depression Scale

The Zung Self-Rating Depression Scale (ZSDS) has a 20-item self-report assessment designed to measure depression symptoms and their levels. The ZSDS scale rate the four common attributes of depression: the pervasive effect, the physiological equivalents, other disturbances, and psychomotor activities (25, 41). The test consists of ten positive and ten negative worded items, scored reversely. Each item is scored on a Likert-type scale with 1 (Never), 2 (Sometimes), 3 (often/most of the time), and 4 (always). The range of the total raw scores is between 20 and 80, both digits inclusive. The raw score was standardized and converted into Depression Index (termed “ZSDS index”) by using the following formula: standard score = INT (1.25 × raw score). Therefore, the total ZSDS index ranges from 25 to 100. A standard score exceeding 50 indicates that individuals suffer from depression symptoms. The standard score is categorized into four levels to determine one's level of depression. Therefore, ZSDS index scores from 25 to 49 (Normal Range), 50 to 59 (Mildly Depressed), 60 to 69 (Moderately Depressed), 70 and above (Severely Depressed) (25). The Arabic translation and validation of the ZSDS scale were done in (43). Due to cultural differences, some thought that question 6, which states “I still enjoy sex,” might offend cultural and religious sensitivities. Accordingly, as stated in (43), the question was adjusted with more appropriate wording, “I enjoy looking at, talking to, and being with attractive women/men,” and the accuracy of the new version was verified (43). The ZSDS questionnaire was prepared in both languages, Arabic and English, side by side, using the Arabic translation as in (43). In this study, Cronbach's α coefficient was 0.869, showing high internal consistency.

### Data Analysis

All analyses were completed using IBM SPSS version 24. Descriptive statistics such as mean, SD, and frequency were used to analyze the demographic data, self-esteem, and depression among university students. The self-esteem level was divided by cut-off points into two levels, low and high, while the depression was divided by the cut-off points into four levels, normal, mild, moderate, and severe. Frequency analysis was performed to investigate the levels of self-esteem and depression among students (gender-wise). Multiple regression analyses and ANOVA were performed, having depression as a regressand and self-esteem and gender as regressors. Logistic regression analysis was performed after dividing the students into two groups, depressed group and non-depressed group, to investigate significant factors and the predictive power for depression. The level of significance was set at *p* ≤ 0.05.

## Results

### Demographics Characteristics

Of the 160 students who participated, nine were excluded as they did not complete the survey questionnaire; for example, they did not answer the RSES questions or ZSDS questions. Thus, the total number who fully participated is 151 students. The demographic characteristic of the respondent is shown in Table 1. Out of the 151 respondents, 74 (49%) were male, and 77 (51%) were female. Most of the respondents, 145 (96%), were 18–24 years old. Regarding their field of study, then the majority, 101 (66.9%), were from the College of Computer and Information Sciences, 34 (22.5%) from the College of Business Administration, the remaining were from Engineering 8 (5.3 %) and Science 8 (5.3%) College.

**Table 1:** Demographic characteristics of the respondents.

**Variable**	**Frequency**	**Percentage**
**Gender**		
Male	74	49%
Female	77	51%
**Age group(years)**		
<18	1	0.66%
18–24	145	96%
25–31	4	2.7%
> 31	1	0.66%
**College**		
Computing and IS	101	66.9%
Business administration	34	22.5%
Engineering	8	5.3%
Science	8	5.3%

### Descriptive Analysis

The mean value for the Rosenberg Self-Esteem Scale (RSES) was 29.4304 (*SD* = 5.190). Looking gender-wise, the mean of the male is 28.7 (SD = 5.29), while the mean of the female was 30.15 (SD = 5.02), indicating that males suffer from low self-esteem more than females. As for the Depression, then the mean of the Zung depression index scale (ZSDS) was 56.705 (SD = 11.764). Again, gender-wise comparison, the male mean is 53.243 (SD = 9.79), while the mean of the female is 60.0325 (SD = 12.577). Females' mean is higher than males, indicating that females suffer more depressive symptoms than males (Table 2).

**Table 2:** Descriptive analysis of RSES scale and ZSDS scale, and gender-wise comparison between levels of self-esteem and depression based on Rosenberg's classifications and Zung's depression index classifications.

**Description of the scale**	**Females** **mean (SD)**	**Males** **mean (SD)**	**Total** **mean (SD)**
Rosenberg self- esteem	30.16 (5.02)	28.68 (5.29)	29.43 (5.19)
Zung depression scale	60.03 (12.58)	53.24 (9.79)	56.71 (11.76)
**Self-esteem and depression level**			
	**Female** **(*****N*** **= 77)**	**Male** **(*****N*** **= 74)**	**Total** **(*****N*** **=151)**
**Rosenberg SES**			
High self-esteem	48 (62.3%)	41 (55.4%)	89 (58.9%)
Low self-esteem	29 (37.7%)	33 (44.6%)	62 (41.1%)
**Zung index scale**			
Normal	14 (18.2%)	24 (32.4%)	38 (25.2%)
Mildly depressed	24 (31.2 %)	33 (44.6%)	57 (37.7%)
Moderately depressed	17 (22.1%)	14 (18.9%)	31 (20.5%)
Severely depressed	22 (28.8%)	3 (4.1%)	25 (16.6%)

### Pandemic, Self-Esteem, and Depression Levels of the Students

The levels of self-Esteem and depression were investigated using the cut-off points. Table 2 illustrates the level of self-esteem among male and female students. Eighty-nine (59%) have high self-esteem among the respondents, while 62 (41%) have low self-esteem. Investigating gender-wise, one can see that both genders have a more significant percentage of high-level self-esteem than low level; in particular, female students have a higher percentage of high-level self-esteem than males. Repeating the same analysis with depression level among students, a gender-wise summary of the result is shown in Table 2. Among the respondents, 57 (37.7%), 31 (20.5%), and 25 (16.6%) experienced “Mild depression,” “Moderate depression,” and “Severe depression.” The table demonstrates that 29% of female students experienced severe depression, which is very high than male students, only 4%.

### Regression and ANOVA Analysis

The Correlation analysis between the Self-Esteem Scale and the Zung depression scale indicated statistically significant strong negative Pearson's correlation (*r* = −0.570, *p* <0.001).

Multiple regression analysis and ANOVA were carried out to investigate if self-esteem statistically significantly predicts depressive symptoms by taking total Rosenberg self-esteem as a predictor and total Zung Depression as a predictant. From Table 3, the result indicated a statistically significant association (*R*^2^ = 0.325, (*F* (1, 149) = 71.825, *p* = 0.001), *B* = −1.034, *b* = −0.570, *t* = −8.475 *and p* < 0.001), in fact, self-esteem was a 32.5% predictor of depressive symptoms among university students.

**Table 3:** Linear regression analysis between self-esteem and depression levels.

**Total Zung depression scale**	**Coefficients (unstandardized)**		**Coefficients** **(standardized)**	***t*** **-ratios**	**Sig.**
	**Slope**	**Std. Error**	**Slope**		
(Constant)	75.80	3.65		20.79	<0.001
Total Rosenberg self-esteem sum	−1.03	0.12	−0.57	−8.48	<0.001

Upon repeating the same test on each gender, the regression result for male *was* (*R* = 0.689, *R*^2^ = 0.475, *F* (1, 75) = 65.2, *p* = 0.001) which is statistically significant, indicating that self-esteem was almost 48% predictor of depressive symptoms among male students. As for female, the result was also statistically significant, and self-esteem was 39% predictor of depressive symptoms (*R* = 0.624, *R*^2^ = 0.390, *F* (1, 72) = 47.92, (*p* < 0.01)), see Table 4.

**Table 4:** Linear regression analysis between self-esteem and depression-gender wise comparison.

**Total Zung depression scale**	**Coefficients (unstandardized)**		**Coefficients (standardized)**	***t*** **-ratios**	**Sig**.
	**Slopes**	**Std. Error**	**Slopes**		
(constant)	89.93	4.61		19.50	<0.001
(Male) Rosenberg sum	−1.28	0.16	−0.69	−8.08	<0.001
(constant)	107.19	6.91		15.52	<0.001
(Female) Rosenberg sum	−1.56	0.23	−0.62	−6.92	<0.001

### Factors Affecting Depression

The dependent variable depression was classified into two groups: a) depression group, having three levels of depressive symptoms (mild, moderate, and severe), b) non-depression group, having depressive symptoms within the normal range. A binary logistic regression analysis was used to investigate the factors directly correlated with depression. The independent variables were Self-esteem, gender, age, and their colleges. The Hosmer-Lemeshow model was appropriate for depression (*p* = 0.815). The variables, Self-Esteem, and gender were found statistically significant at the given level of significance for this model. It was found that Self-Esteem and gender were correlated with depression Table 5, while age and their colleges were not correlated, thus not included in the table. When self-esteem is high, the depression level is 0.168 times lower, while in the case of gender, it was found that in males, the depression level was 0.380 times lower than in females.

**Table 5:** Factors affecting depression.

**Variables**	**B**	**SE**	**WALD**	**Exp (B) OR**	**95% CI**	***P***
Self-esteem (ref. high self-esteem)	−1.785	0.495	12.983	0.168	0.065–0.443	0.000
Gender (ref. Male)	−0.967	0.411	5.543	0.380	0.170–0.850	0.019

## Discussion

Students everywhere agonize from psychological distress, mainly self-esteem, depression, stress, and anxiety, due to academic pressure and concern about the future (44). The presence of the pandemic is an added factor, as many students feel augmented stress levels, anxiety, and depressive symptoms as a consequence of changed teaching method and reservation of university education, technological concerns of online courses, and future employment; all these influences were witnessed in universities around the globe (11). Several studies have discussed the students' psychological wellbeing during the COVID-19 pandemic (45). For example, a study conducted in the USA (46) indicated a 71% increase in stress and anxiety level in students due to the COVID-19 outbreak.

The present study aimed to examine the self-esteem and depression level (gender-wise) among the university students in Riyadh during the COVID-19 outbreak and investigate whether there is a positive or negative correlation between self-esteem and depression and explore factors affecting depression.

The result revealed that three fourth (75%) of the university students suffer different levels of depression, and half of those (37.5%) suffer from moderate to extreme levels of depression, which is quite a high percentage but is consistent with the research result in the USA, which showed that 44% of the participants in the USA showed a high level of depressive thoughts (46). As for self-esteem, 41% of the university students in Riyadh suffer from low self-esteem. Comparing gender-wise, female students have a more significant percentage of high-level self-esteem than males; this is consistent with Al-Qahtani et al. (47), where he demonstrated that females in Saudi Arabia have higher self-esteem levels.

Health research broadly studied the association between self-esteem and depression over the past era (27). Our study indicated a strong negative statistically significant correlation between self-esteem and depression. A total of 41% of students experienced low self-esteem, 38% females and 45% males. Multiple regression analysis findings displayed that low self-esteem is an influencing factor for having a higher level of depression, which is consistent with the research result in (34), which showed that low self-esteem contributed significantly to depression; it was reported that the likelihood of the students with low self-esteem was found to have four times higher depressive symptoms (34).

A closer look at the depression levels among university students, our study demonstrated that 38%, 20%, and 17% suffer from “mild,” “moderate,” and “extreme” levels of depression, respectively, during the pandemic. The binary logistic regression analysis has indicated that having a higher level of self-esteem reduces the chances of getting depressive symptoms by ~17%; in addition, the depression in male students was 38% lower than in females. The univariant analysis showed that low self-esteem contributed to high depression level risks, as 29% of females suffered from extreme levels of depression, which is very high compared to males, which was only 4%. Although females showed higher percentages of high Self-Esteem than males, we argue that there is an association between females and emotionally unstable personalities, as they are more prone to it (48). Females are more vulnerable than males to disasters (49).

Our findings indicated high depressive symptoms in students during the pandemic, particularly in females, which is not shocking. Moreover, it supports previous studies, showing that Vietnamese female students had a higher percentage of depression than male students (34). In the COVID-19-time period, Chinese females experienced more anxiety than males (50). This is largely attributable to the inner dynamics of Chinese mainland health system (51). It is largely associated with the national healthcare spending for non-communicable diseases inclusive of mental disorders (52). Saudi Arabia as the leading Gulf countries economy is meaningful to compare with the underlying hidden patterns in most rapidly developing BRICS Emerging Markets extending up to 2025 (53) and 2030 (54).

## Conclusion

The findings of this research showed that COVID-19 had been a disastrous experience. The results reveal that depression of different levels is among three fourth of the university students in Saudi Arabia. Half of them had moderate to extreme levels of depressions during the pandemic, which is higher than the predicted depression level among the general population. It should be noted that those students are young, most of their ages are between 18 and 24 years; some studies noted that psychological distress, stress, and anxiety are higher in the younger age group during the COVID-19 pandemic (22), which is consistent with our result. Furthermore, the rate of depressive symptoms in female students is higher than the males. As for self-esteem, 40% of the student experience low self-esteem, and low self-esteem makes students more prone to developing depressive symptoms. On the other hand, having a higher level of self-esteem reduces the chances of getting depressive symptoms by 17%.

Based on the findings of this study, there is a need for instant consideration and support for students. We need to search for protentional managing strategies that have been proven effective during a pandemic (55). The results of our study might guide policymakers to develop risk control procedures as part of their policy for the future to contain future pandemics (56). Furthermore, training the students to shift their mindset about the educational experience into a positive side, having stress-related growth, is necessary (57). An adaptive mindset can help students familiarize themselves with the new learning methods.

## Strength and Limitation of Our Study

The study's strength is that it was conducted in the largest city of Saudi Arabia which is Riyadh. Furthermore, the Rosenberg Self-Esteem Scale and Zung Self-Rating Depression Scale are well-known and reliable measures.

The limitation of the study is using a convenient sampling method; thus, the sample was not randomly selected from all university students in Saudi Arabia. Never the less, the sample is a good representative for our study reflecting students' self-esteem and depression levels in Saudi Arabia, as it was conducted during the virtual teaching method in Saudi Arabia and from two reputed Universities in Riyadh.

## Data Availability Statement

The raw data supporting the conclusions of this article will be made available by the authors, without undue reservation.

## Ethics Statement

This study was approved by Prince Sultan University's Institutional Review Board (IRB) (Reference Number: PSU IRB-2021-03-0077).

## Informed Consent

Informed consent was obtained from all individual participants included in the study.

## Author Contributions

FA: writing the original manuscript, draft, data collection, data analysis, writing the paper, and review and editing. HK: data analysis and review and editing. AA: data collection, review and editing, and supervising. AY and MJ: review and editing. All authors read and approved the final version of the paper.

## Conflict of Interest

The authors declare that the research was conducted in the absence of any commercial or financial relationships that could be construed as a potential conflict of interest.

## Publisher's Note

All claims expressed in this article are solely those of the authors and do not necessarily represent those of their affiliated organizations, or those of the publisher, the editors and the reviewers. Any product that may be evaluated in this article, or claim that may be made by its manufacturer, is not guaranteed or endorsed by the publisher.
